# Tubular Micro/Nanomotors: Propulsion Mechanisms, Fabrication Techniques and Applications

**DOI:** 10.3390/mi9020078

**Published:** 2018-02-13

**Authors:** Fengjun Zha, Tingwei Wang, Ming Luo, Jianguo Guan

**Affiliations:** State Key Laboratory of Advanced Technology for Materials Synthesis and Processing, International School of Materials Science and Engineering, Wuhan University of Technology, Wuhan 430070, China; zhafj159@whut.edu.cn (F.Z.); 315387183@whut.edu.cn (T.W.)

**Keywords:** tubular Micro/Nanomotors, propulsion mechanisms, fabrication techniques, applications

## Abstract

Micro/nanomotors are self-propelled machines that can convert various energy sources into autonomous movement. With the great advances of nanotechnology, Micro/Nanomotors of various geometries have been designed and fabricated over the past few decades. Among them, the tubular Micro/Nanomotors have a unique morphology of hollow structures, which enable them to possess a strong driving force and easy surface functionalization. They are promising for environmental and biomedical applications, ranging from water remediation, sensing to active drug delivery and precise surgery. This article gives a comprehensive and clear review of tubular Micro/Nanomotors, including propulsion mechanisms, fabrication techniques and applications. In the end, we also put forward some realistic problems and speculate about corresponding methods to improve existing tubular Micro/Nanomotors.

## 1. Introduction

Motion has been essential for life over millions of years of evolution and is a common phenomenon in every living cell and organism. Microscopic biological motors [1,2,3] and machines are involved in numerous cellular or organismal activities, such as ATP synthesis, DNA replication and protein synthesis in cells, as well as the free locomotion of living creatures from ants to blue whales. Inspired by the biological motors and machines of nature, considerable efforts have been devoted to developing self-propelled artificial Micro/Nanomotors that can mimic these amazing functions of natural systems [3,4,5]. Artificial Micro/Nanomotors are machines at a micro/nanometer scale that are able to transduce other forms of energy from their surrounding into autonomous movement [6,7]. They may bring about revolutionary changes in micro/nanoengineering, environment treatment [8,9,10], biomedicine [11,12,13,14], etc.

Thanks to the advances in the nanotechnology over the past decades, Micro/Nanomotors with various geometries have been developed, such as rods [15,16,17,18,19], Janus spheres [20,21,22,23,24,25,26,27,28], tubes [29,30,31,32,33,34,35,36] and so on. The systematical and in-depth studies demonstrate that the geometry of Micro/Nanomotors has a huge impact on their performance in terms of propulsion mechanisms, motion behaviors and subsequent applications [3]. For example, the Au‒Pt bimetallic nanorods synthesized by Sen and co-workers [15] are capable of moving autonomously in a solution of H_2_O_2_ with a speed of 2–10 body lengths per second. The motion of such bimetallic nanorods follows a self-electrophoresis propulsion mechanism, which is susceptible to the ionic strength of the solution. For Janus spheres Micro/Nanomotors using Pt as a catalyst, the propulsion mechanisms generally vary with their particle sizes [37]. At a small size, they move in a self-phoresis propulsion mechanism, while those with a relative large size may be propelled by bubble recoiling. The latter have a relatively strong driving force and thus move faster than the former. Compared to bimetallic nanorods and Janus spheres, tubular Micro/Nanomotors have a unique hollow structure, providing a confined space for chemical/biochemical reactions or physical transformations. This advantage enables tubular Micro/Nanomotors to be easily propelled by a strong thrust of bubbles [31,32,38]. For instance, the bilayer poly (3,4-ethylenedioxythiophene) (PEDOT)/Pt tubular micromotors fabricated by Wang and co-workers [32] can move at a speed of over 1400 body lengths per second and only require a very low H_2_O_2_ concentration. The speed of tubular Micro/Nanomotors can be further improved to over 158,000 body lengths per second by using ultrasound as an energy source. These bubble-propelled tubular Micro/Nanomotors can locomote efficiently in high ionic-strength media and relevant biological fluids. By combining the efficient autonomous motion with the large surface areas, tubular Micro/Nanomotors offer great promise for wide important applications, ranging from water remediation [8,9], sensing [39,40] to active drug delivery [41,42] and invasive surgery [43,44].

Tubular Micro/Nanomotors were named as microjets and microrockets for the first time [29]. Up to date, various tubular Micro/Nanomotors have been developed including not only catalytically propelled tubular Micro/Nanomotors but also those propelled by chemical/biochemical reactions, external fields and motile microorganisms. The recent review by Mei and his colleagues, which we noted almost at the same time of our submission, mainly focused on catalytically propelled tubular Micro/Nanomotors [45]. Herein, we attempt to present a comprehensive review on the state-of-the-art of tubular Micro/Nanomotors in balance. After briefly illustrating the dependence of the motion behaviors of Micro/Nanomotors on their morphologies, we introduce in Section 2 the relationship between propulsion mechanisms of tubular Micro/Nanomotors and their hollow structures with typical examples and discuss the corresponding motion behaviors. Then, we summarize in Section 3 the fabrication techniques of tubular Micro/Nanomotors, which mainly include rolled-up and template-assisted methods. In Section 4, we highlight the recent developments of environmental and biological applications based on those tubular Micro/Nanomotors. Finally, we also envision the current challenges and future prospects of tubular Micro/Nanomotors before some concluding remarks. We hope this article will provide readers with useful information and insights and promote the further development of tubular Micro/Nanomotors.

## 2. Propulsion Mechanisms

The key point to design self-propelled artificial Micro/Nanomotors is to construct an asymmetric field across micro/nanostructures to break the pressure symmetry. Thus far, a variety of asymmetric fields, such as a local electric field, concentration gradients, surface tension gradients, temperature gradients and bubbles, have been employed to drive Micro/Nanomotors. Compared to Micro/Nanomotors with bimetallic nanorods and Janus structures, tubular Micro/Nanomotors possess unique inner hollow structures. This enables chemical/biochemical reactions or physical transformations to be confined in the inner cavity of the tubes. As a result, tubular Micro/Nanomotors commonly possess a relatively strong driving force. In this section, we illustrate the different propulsion mechanisms of tubular Micro/Nanomotors and discuss the corresponding motion behaviors such as driving force or lifetime.

**Figure 1:**
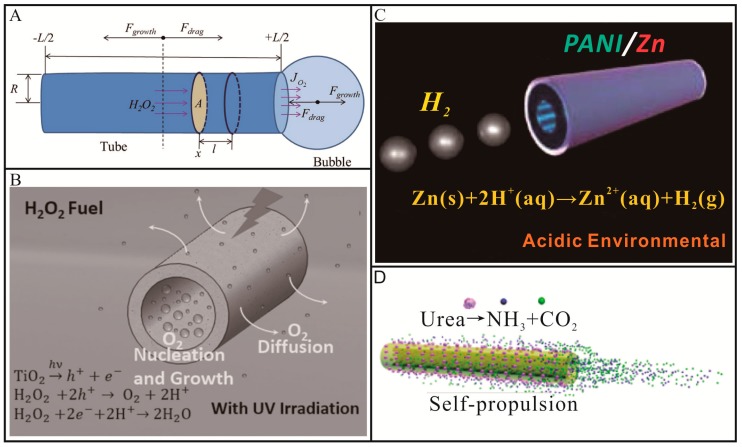
Representative examples of chemically/biochemically propelled tubular Micro/Nanomotors. (**A**) Schematic illustration of tubular catalytic micromotor propelled by O_2_-bubble ejection in an aqueous H_2_O_2_ solution (adapted from Reference [46]); (**B**) Schematic diagram of photocatalytic reaction propelled motion of TiO_2_ micromotor in an aqueous H_2_O_2_ solution under the irradiation of UV light (adapted from [50]); (**C**) Schematic diagram of motion for tubular polyaniline (PANI)/Zn micromotor in an acidic environment (adopted from [36]); (**D**) Schematic illustration of the motion of urease-conjugated SiO_2_ tubular micromotor (adapted from [56]).

Most tubular Micro/Nanomotors are propelled by (photo)catalytic/chemical reactions [29,30,31,32,36,46,47,48,49,50,51,52,53,54,55] occurring in the inner hollow structures. For this type of tubular Micro/Nanomotors, a catalyst including noble metals and semiconducting oxides, or active metals should be incorporated in the body of micro/nanotubes to trigger chemical/biochemical reactions in the presence of fuels, such as H_2_O_2_. As shown in Figure 1A, noble metals, such as Pt, can decompose H_2_O_2_ into H_2_O and O_2_ at the inner wall of tubular Micro/Nanomotors [46,47,48,49]. The resulting O_2_ molecules would nucleate, grow into bubbles, diffuse and finally burst or break away from one open end of tubular Micro/Nanomotors. Once a bubble was ejected from one open end of the tube, the tube started to move in the opposite direction and the symmetry of bubble ejection was broken. In this way, the open end with the first bubble ejection would always act as bubble exhaust nozzle and the other open end of the tube would serve as the feeding inlet of aqueous fuels. For tubes with different structures (cone or cylinder), the situation will be different. When the tubes possess conical structures, the large opening end would always serve as bubble exhaust nozzle and the movement direction would be toward the small opening end [30]. While in the case of cylindrical structures, the two opening ends can both serve as bubble exhaust nozzle. Meanwhile, the influence of the tube length cannot be ignored. When the tube is too long, the first ejected bubble could not move the tube. In this case, the symmetry for the bubble ejection does not break [50]. Our group demonstrated a TiO_2_-based tubular micromotor [50], as shown in Figure 1B. When irradiated by ultraviolet (UV) light, TiO_2_ with a band gap of 3.2 eV would excite electron–hole pairs according to Equation (1), these photo-generated electrons and holes could react with H_2_O_2_ to produce O_2_ and H_2_O described as Equations (2) and (3). When the inner diameter and length of the microtube were tuned, the O_2_ molecules would preferentially nucleate and grow into bubbles on the inner concave surface rather than on the outer surface, resulting in strong propulsion of the micromotor. The motion behaviors and speed of this micromotor can be reversibly, wirelessly and remotely controlled at will with an ultrafast response rate (less than 0.2 s) by regulating “off-on” switch and intensity of UV irradiation. Using the same propulsion mechanism but alternative fuels of acids, Wang and co-workers [36] reported a polyaniline (PANI)/Zn bilayer tubular micromotor. In a strongly acidic solution, H_2_ bubbles will be produced in the inner cavity of microtube through a spontaneous redox reaction (Equation (4)), leading to ultrafast propulsion of tubular micromotors (Figure 1C). Such acid-powered tubular micromotors are able to perform in extreme environments. However, owing to the consumption of Zn, these acid-powered tubular micromotors will stop run within a few minutes, which will hinder their practical applications. Sánchez and co-workers [56] reported a ultrasmall tubular SiO_2_ nanomotor using urea as fuels. This nanomotor was propelled by the turnover of urea substrate triggered by urease according to Equation (5). As shown in Figure 1D, the reaction products, NH_3_ and CO_2_, were formed inside the nanotubes, generating internal flows that extent into the external space via the tube opening. Both the inside and outside urease contributed to the motion of the nanomotors. No visible bubbles were observed for this nanomotor, which held great promise for use in biomedical fields. Compared to bubble-propelled micromotors, the driving force of these nanomotors is relatively weak.

TiO_2_ + *hv* → *h*^+^ + *e^−^* (1)

H_2_O_2_ + 2*h*^+^ → O_2_ + 2H^+^ (2)

H_2_O_2_ + 2*e^−^* + 2H^+^ → 2H_2_O (3)

Zn(s) + 2H^+^(aq) → Zn^2+^(aq) + H_2_(g) (4)

Urea → NH_3_(aq) + CO_2_(aq) (5)

**Figure 2:**
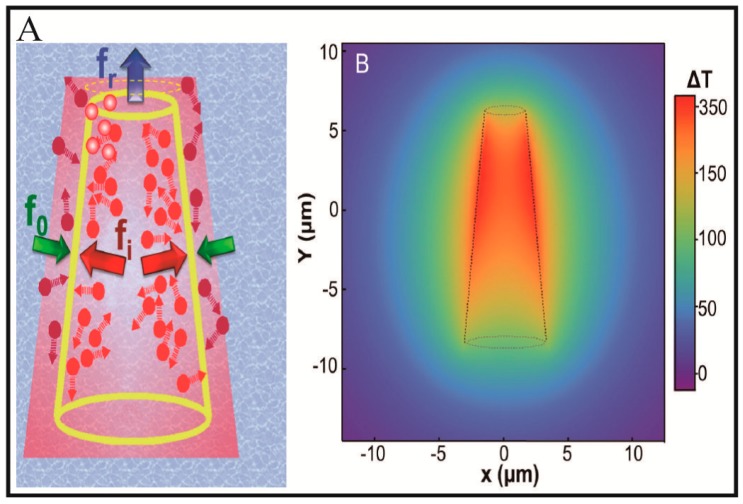

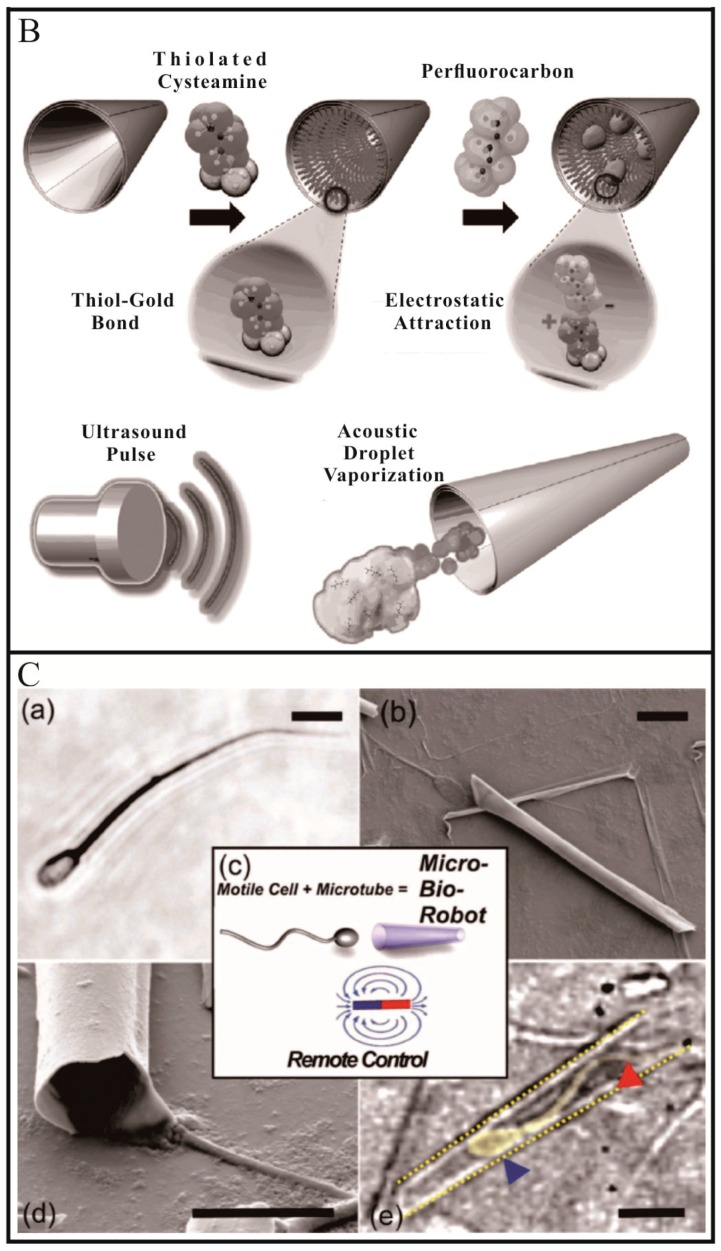
Representative examples of external field or motile microorganism-propelled tubular Micro/Nanomotors. (**A**) Schematic diagram of Au nanoshells powered by self-thermophoresis upon exposure to a NIR laser (adapted from [57]); (**B**) Schematic illustration of acoustic droplet vaporization and propulsion of PFC-loaded micromotors triggered by an ultrasound pulse (adapted from [58]); (**C**) A bio-hybrid tubular micromotor combining a single motile sperm cell with a rolled-up microtube (adapted from [61]).

Tubular Micro/Nanomotors can also be propelled by external fields [57,58,59,60] or motile microorganisms [61,62,63,64]. External field-induced physical transformations can be confined in the inner cavity of the micro-/nanotubes. Wu and co-workers [57] demonstrated a near-infrared (NIR) light-driven polymer multilayer tubular micromotor. Under the irradiation of an NIR laser, the Au shell with a strong plasmon resonance absorption in the NIR region created local temperature gradients around the wall of the microtube, as depicted in Figure 2A. Owing to the asymmetric structure of the microtube, the temperature in the inner surface will be higher than in the outer surface. As a result, two thermophoretic forces (*f*_i_ > *f*_o_) perpendicular to the inner and outer surface of the microtube were generated. Due to the conical structure of the microtube, the net resultant force (*f*_r_) would face toward the front small-opening along the longitudinal axis of the microtube. This net resultant force pushed the micromotor to move fast toward, in the direction of the front small-opening and roughly maintains a linear trajectory. Wang and co-workers [58] reported a superfast ultrasound-propelled tubular micromotor. As depicted in Figure 2B, the thiolated cysteamine was first modified to the inner Au layer through thiol-Au bond, allowing for electrostatic binding of the anionic perfluorocarbon (PFC) droplets. The PFC droplets can be vaporized by ultrasound to produce ejected gas bubbles, propelling the microtube as other bubble-propelled micromotors. This tubular micromotor can travel at remarkably high average velocities at over 158,000 body lengths per second, providing a strong thrust to deeply penetrate and deform tissues. However, the microtube will stop once it runs out of PFC droplets, suggesting that the propulsion only lasts for a short time. Motile microorganism can be trapped into the inner cavity of a microtube when they have a similar size. Sánchez and co-workers [61] reported a sperm-propelled bio-hybrid micromotor. As shown in Figure 2C, a sperm cell was trapped into the tube cavity of a microtube. The as-prepared bio-hybrid micromotor can be propelled by the sperm cell. By incorporating a magnetic component into the microtube, the direction of the bio-hybrid micromotors can be controlled by external magnetic fields. These sperm-propelled bio-hybrid tubular micromotors promise the biocompatibility, flexibility and untethered operation.

To date, various energy sources, such as chemical/biochemical reactions, external fields and motile microorganisms, have been employed to propel tubular Micro/Nanomotors. The confinement effect of the hollow structures enables the driving force of tubular Micro/Nanomotors to be much stronger than other Micro/Nanomotors with different geometries.

## 3. Fabrication Techniques

Fabrication techniques play a vital role in the realization of the practical applications of tubular Micro/Nanomotors, as each application needs special functional units. Over the past decade, a large number of fabrication techniques have been developed to prepare tubular Micro/Nanomotors. Each of these techniques has its own advantages and limitations in terms of scalability, precision, cost, or device size. In this section, we summarize different types of fabrication techniques and divide them into two parts: rolled-up method and template-assisted method.

### 3.1. Rolled-Up Method

Rolled-up method has been widely used for the design of tubular Micro/Nanomotors, which is generally based on strain engineering [29]. By incorporating an engineered strain gradient in the deposited membranes, the membranes can form into desired structures when released from the substrate. Thus far, a variety of materials or materials combinations have been used for this fabrication method.

**Figure 3:**
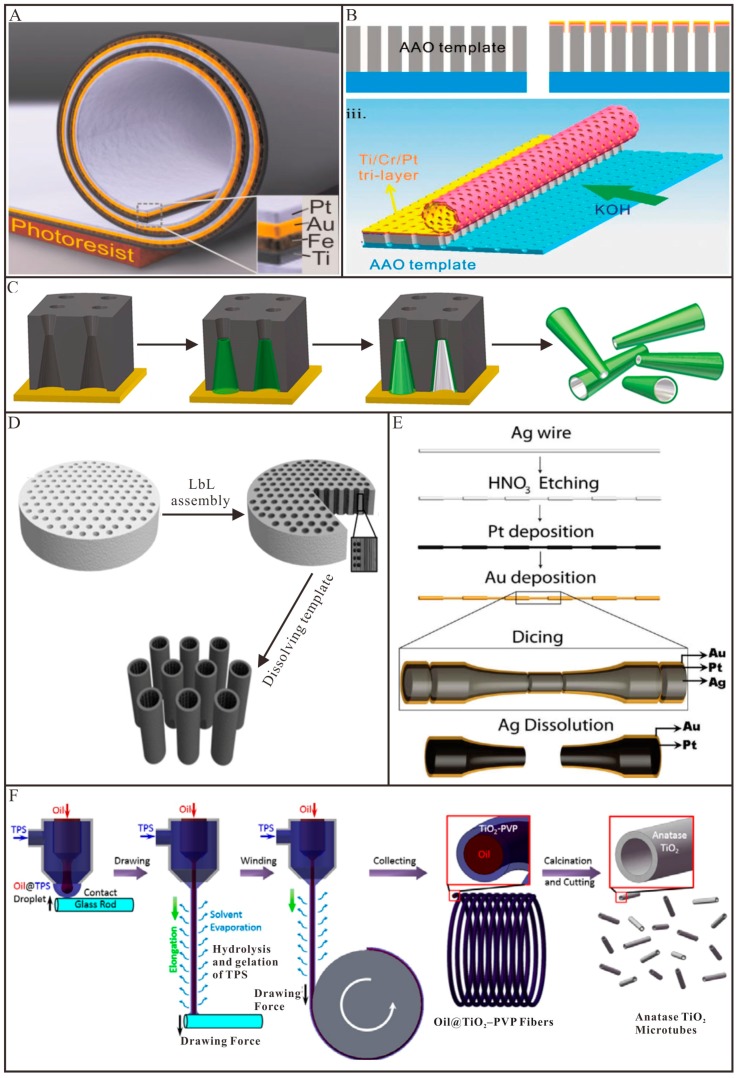
Representative examples of the fabrication techniques of tubular Micro/Nanomotors. (**A**) Schematic diagram of a rolled-up tubular micro/nanomotor consisting of Pt/Au/Fe/Ti multilayers on a photoresist sacrificial layer (adapted from [30]); (**B**) Three-dimensional schematic illustration of the fabrication method for the nanoporous tubular micromotors used anodic aluminum oxide (AAO) as a sacrificial template (adapted from [68]); (**C**) Preparation of bilayer PANI/Pt tubular micromotors using polycarbonate membranes (adapted from [31]); (**D**) Fabrication of polyelectrolyte multilayer tubular nanomotors. Black dots and vertical stripes represent Pt nanoparticles and polyelectrolyte multilayers, respectively (adapted from [75]); (**E**) Silver wire template-assisted layering approach for preparation of tubular micromotors (adapted from [76]); (**F**) Schematics to demonstrate the preparation of the TiO_2_ tubular micromotor by a dry spinning method and subsequent calcination (adapted from [50]).

Mei and co-workers fabricated several tubular micromotors by using rolled-up method [30,35,65]. As shown in Figure 3A, a photoresist layer was first deposited on the substrate and served as a sacrificial layer [30]. Subsequently, different metals were deposited in order to produce a pre-stressed, multimetallic thin membrane. By selectively removing the photoresist layer with acetone, the thin membrane spontaneously rolled up into a tubular Micro/Nanomotors. The Pt inner layer was served as the catalyst, while the Fe layer was used for magnetic guidance. The diameter and length of the tubular micromotors can be controlled by deposition parameters (e.g., film thickness and strain) and lithographically predefined patterns, respectively. Apart from metals, other materials such as semiconductors, oxides, magnetic materials and polymers can also be used to rolled-up into tubular structures [66,67]. However, the above-mentioned rolled-up methods can only be fit to fabricate few tubular Micro/Nanomotors, when tons of motors are needed, the cost of the harsh preparation conditions (e.g., clean room) will be a huge challenge. Thus, great efforts have been devoted to simplifying the rolled-up process and reducing its cost. Li and co-workers [68] utilized anodic aluminum oxide (AAO) membranes as a sacrificial template to fabricate rolled-up tubular micromotors. As illustrated in Figure 3B, pre-strained Ti/Cr/Pt metallic tri-layers were subsequently deposited on the AAO membranes by e-beam evaporation. After metal deposition, the upper metallic tri-layers were divided into quadrate patterns with a size of 10–30 µm using a fine mechanical scratching process. Finally, potassium hydroxide (KOH) solution was used to selectively dissolve AAO sacrificial templates, leading to the release of the patterned metallic tri-layers. The intrinsic stress in the Ti/Cr/Pt tri-layers made them automatically roll up into microtubular structures. The produced microtubes exhibited higher velocities compared to those with a smooth platinum surface. Yao and co-workers [69] fabricated a tubular micromotor which was scrolled from graphene oxide (GO). In this case, GO has two functions: acting as a support for the metallic layers and providing an easily cleavable interface between the substrate and the metallic layers. Because of material strain and weak bonding between GO layers, microscrolls with GO on the outside and Pt at the inner surface were spontaneously formed upon sonication. The diameter can be modulated by changing the thickness of the deposited metal layers. Pumera and co-workers [48] reported a similar method to fabricate rolled-up tubular micromotors by using fruit tissue cells as the support for the deposited metal layers. The as-prepared tubular micromotors exhibited excellent mobility in the presence of H_2_O_2_. The same group [70] demonstrated the formation of Pt microtubes by using a transmission electron microscopy grid as templates. In this work, selective removal of the poly (methyl methacrylate) (PMMA) sacrificial layer under the deposited Pt layer or H_2_O_2_-assisted lift-off of the Pt layer deposited directly on a glass substrate were used to fabricate Pt microtubes. These clean room-free methods are simple and cheap; however, they cannot precisely control the size and morphology of the fabricated tubular micromotors.

To date, various rolled-up methods have been developed to fabricate tubular micromotors with different components. In addition, rolled-up technology has been used to demonstrate the first tubular micromotor with soft reconfiguration and it can also integrate ultra-compact electronic functionality like antennas [71] or integrated circuitry [72] into microtubes. Overall, future efforts should be provided to simplify the fabrication procedure of rolled-up methods.

### 3.2. Template-Assisted Method

Template assembly is a method to deposit a variety of materials into or onto the templates to form a multilayer tubular structure. Then, get rid of corresponding templates, so the tubular Micro/Nanomotors can be released. This kind of fabrication technique is considered to be pretty easy, low-cost and promises industrial process. Compared with rolled-up method, template-assisted method can prepare Micro/Nanomotors with smaller sizes. According to the position of the template, template-assisted method can be classified into external template-assisted method and internal template-assisted method.

#### 3.2.1. External Template-Assisted Methods

External template-assisted methods prepare tubular Micro/Nanomotors within a template [31,32,36,60,73,74,75]. A membrane, such as track-etched polycarbonate membranes and porous alumina membranes, is commonly utilized as a template in external template-assisted methods. Each pore of the membrane can be utilized as a separated reactor to synthesize desired tubular micro-/nanomotor with different size and composition. Owing to the monodisperse diameters and large pore densities in the membrane, uniform tubular Micro/Nanomotors can be mass produced. Thus, these methods are powerful and relatively low-cost for preparing tubular Micro/Nanomotors. External template-assisted methods discussed here mainly include membrane template-assisted electrodeposition and layer-by-layer (LbL) assembly.

Membrane template-assisted electrodeposition is the most commonly used methods to fabricate tubular Micro/Nanomotors. For example, Wang and co-workers [31] developed a simplified method for fabricating tubular micromotors within the conically shaped pores of a polycarbonate membrane. As shown in Figure 3C, an Au film was first sputtered on one side of the polycarbonate membrane to serve as the working electrode. Aniline monomers were then deposited, followed by sequential deposition of Pt. Finally, the Au film and the polycarbonate membrane were removed by mechanical polishing and chemical etching, respectively. The resulting bilayer PANI/Pt micromotors were conical in shape with lengths of several micrometers and diameters depending on the pore size of the membrane template. These tubular micromotors exhibit ultrafast speed and require a very low concentration of H_2_O_2_ fuel. Following this work, the same group [32] investigated the composition and electropolymerization conditions of polymer-based tubular micromotors to realize optimization. PEDOT-based microtubes were found to provide more reproducible yields and consistent morphology. The most favorable condition was the use of a low monomer concentration together with the proper amount of surfactant and appropriate analyte. Instead of taking advantage of Pt-catalyzed decomposition of H_2_O_2_ as a source of power, Wang’s group [36] described tubular PANI/Zn micromotors using acid in the environment as fuel. A layer of Zn was electrodeposited to serve as the inner wall of the microtubes after the electropolymerization of PANI. Except for polymer-based tubular micromotors, complete metallic micro/nanotubes can also be prepared via membrane template-assisted electrodeposition. Pumera and co-workers [73] successfully fabricated bimetallic Cu/Pt microtubes and proposed the use of widely available colloidal graphite ink instead of deposited metal backings in the setup of the electroplating cells to simplify the procedure. A striped metallic nanotube with a diameter of ~300 nm was prepared by electrodeposition employing conductive silver ink on the anodized aluminum oxide (AAO) membrane template together with aluminum foil as the working electrode.

Membrane template-assisted LbL assembly offers an easy and inexpensive method for fabricating multilayer tubular Micro/Nanomotors. The multilayer tubular structures are assembled by oppositely charged materials within the pores of membrane via electrostatic interaction. By incorporating catalysts, such as Pt nanoparticles or catalase, into the wall of multilayer tubular structure, the formed multilayer tubular structure can be propelled in H_2_O_2_ aqueous solutions. For example, He and co-workers [75] illustrated well-defined polymer multilayer tubular nanomotors by using membrane template-assisted LbL assembly. As depicted in Figure 3D, two oppositely charged biodegradable materials, chitosan (CHI) and sodium alginate (ALG), were alternatively absorbed on the nanopores of polycarbonate membrane and followed by the assembly of Pt nanoparticles into the template pores. Nanotubes with catalytic Pt nanoparticles on the inner wall can be obtained upon dissolution of the membrane. Such multilayer tubular Micro/Nanomotors can encapsulate various materials, such as small organic molecules, inorganic compounds, macromolecules and colloids, thus offer possibilities for directed drug delivery.

#### 3.2.2. Internal Template-Assisted Methods

In contrast to external template-assisted methods, internal template-assisted methods prepare tubular Micro/Nanomotors on the outside of a template. Wang and co-workers [76] reported an approach to prepare conical tubular micromotors using etched silver wire as a template. As imaged in Figure 3E, after sequential electrodeposition of Pt and Au layers onto the surface, Ag wires were diced into microcones of the desired length. With nitric acid treatment, the Ag wire template was etched and the Pt/Au bilayer on the wire surface became conical microtubes. The diameter of the larger opening is the same as that of the Ag wires (50 µm). However, this approach is not suitable for mass production and the velocity of microtubes fabricated by this method is relatively low. By using Ag nanowires as templates, Sánchez and co-workers [56] fabricated an ultrasmall tubular silica nanomotor. A thin layer of silica was first grown on the surface of Ag nanowires by sol-gel chemistry. The silica coated Ag nanowires were then broken down into shorter segments by sonication. After removal of the Ag nanowires templates by etching in aqua regia, the silica nanotubes were obtained and functionalized with amine group by grafting. Urease was further conjugated onto the surface of the silica nanotubes via covalent conjugation. The as-prepared tubular nanomotors can be propelled by using biofriendly urea as a fuel without visible bubbles. Our group [50] reported a coaxial spinning method to fabricate light-driven tubular TiO_2_ micromotors. As shown in Figure 3F, the TiO_2_ precursor solution (TPS) and the heavy paraffin oil were added to two separated syringes connected to a coaxial spinneret. The TiO_2_ precursor solution was used as an outer water phase and the heavy paraffin oil (Oil) was used as an inner oil phase. Then the Oil@TPS droplet was drawn into a liquid Oil@TPS jet by pulling the glass rod, which was previously contacted with the droplet, away from the spinneret. With the evaporation of solvents as well as the hydrolysis and gelation of TiO_2_ precursor, the liquid Oil@TPS jet was solidified into Oil@TiO_2_–PVP fiber. By the decomposition of the organic components with calcination, the collected Oil@TiO_2_–PVP fibers were finally transformed into the TiO_2_ hollow fibers. The TiO_2_ hollow fibers were then cut into short microtubes.

Above all, template assembly is an easily accessible method and can be pretty cheap and fast. However, this technique inevitably has some shortcomings. For example, the size and shape of the resulting tubes are limited to presented template pores. In addition, the released tubular Micro/Nanomotors are difficult to be complete and uniform after dissolving the template.

## 4. Tubular Micro/Nanomotors towards Practical Applications

Tubular Micro/Nanomotors can effectively convert diverse energy sources into fast movement. In addition, the large surface area can be used to functionalize with various functional units. Thus, tubular Micro/Nanomotors have shown great potential applications, such as environmental and biomedical applications. For environmental applications, the bubble-propelled tubular Micro/Nanomotors by using H_2_O_2_ as fuels are widely explored. For certain biomedical applications, especially in vivo, the biocompatibility of the energy sources should be taken into account. While still in an early stage, attempts to explore environmental and biomedical applications of tubular Micro/Nanomotors are extremely active and encouraging. In this section, we present a detailed description of tubular Micro/Nanomotors towards practical applications in water remediation, sensing, drug delivery and precise surgery, which displays prominent superiorities to traditional methods.

### 4.1. Water Remediation

Water contaminations, such as organic compounds and heavy metals, are a serious risk to the public health and other life forms on earth. Current advances in nanotechnology have increased the interest of finding useful nanomaterials and nanotools for the fast and efficient removal of pollutants from water. Compared with their static counterparts, tubular micro/nanomotor hold great promise for water remediation. To date, a variety of tubular Micro/Nanomotors have been fabricated to degrade or remove pollutants from waste water.

**Figure 4:**
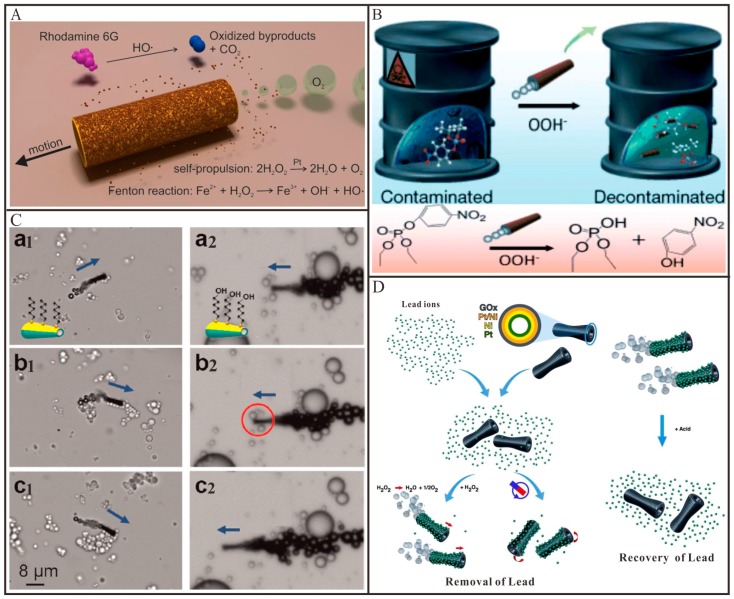
Representative examples of tubular Micro/Nanomotors for water remediation. (**A**) Schematic process for the degradation of polluted water (rhodamine 6G as a model contaminant) into inorganic products by multifunctional micromotors (adapted from [8]); (**B**) Illustration of a micromotor-based accelerated oxidative decontamination of organophospate nerve agents (adapted from [9]); (**C**) C6-SAM-modified micromotors with different head functional groups that can (left) or cannot (right) pick up small olive oil droplets (adapted from [34]); (**D**) Schematic images of GO-micromotors based approach for lead decontamination and recovery (adapted from [82]).

Sánchez and co-workers [8] demonstrated the first example that utilized the self-propelled tubular micromotors to degrade organic pollutants in aqueous solutions. The tubular Fe/Pt bilayers micromotors were prepared by rolled-up technique. As shown in Figure 4A, these tubular micromotors combined two functions: the inner Pt layer was used to decompose H_2_O_2_ into O_2_ and H_2_O for the self-propulsion and the outer Fe layer was used for in situ generation Fe^2+^ ions to trigger the Fenton reaction (Equation (6)). The hydroxyl radical (•OH) generated from Fenton reaction was employed to degrade the organic pollutants. As a result, the degradation efficiency of the model pollutant (Rhodamine 6G) by these self-propelled tubular micromotors was 12 times faster than that of their static counterparts. Almost in the meanwhile, Wang and co-workers [77] demonstrated that the tubular PEDOT/Pt micromotors could accelerate the decontamination of organophosphate nerve agents in waste water (Figure 4B). Similar to the above-mentioned work, H_2_O_2_ has double functions, acting as a fuel to propel the tubular micromotors that contributed to an efficient fluid mixing and serving as an oxidizing reagent for the in-situ generation OOH^−^ nucleophiles in the presence of peroxide activator (NaHCO_3_ or NaOH). Under mild quiescent conditions, the organophosphate nerve agents were oxidized into para-nitrophenol by OOH^−^ nucleophiles and a decontamination of 100% could be achieved with sufficient micromotors. However, when the same mixture with organophosphate pollutants and H_2_O_2_ reacted for the same duration, in the absence of the micromotors, the oxidation process was not observed in the aqueous solution. In addition to Pt as a catalyst, Schmidt and co-workers [78] explored a Pd-based catalytically tubular micromotor for efficient water cleaning. This tubular micromotor can decompose the target pollutant (C_6_H_5_NO_3_) into nontoxic byproducts (C_6_H_7_NO) and H_2_ bubbles, as described in Equation (7) and thus propel the microtube forward. The improved intermixing ability of the micromotors caused a 10 times faster degradation, as compared to their static counterparts. Moreover, taking the photocatalytic activity of TiO_2_ into account, Wang and co-workers [79] reported an internally/externally bubble-propelled photocatalytic tubular nanomotor for the photodecomposition of organic pollutants. Two kinds of tubular nanomotors were fabricated by selective deposition of Pt nanoparticles on the inside or outside surface of TiO_2_ nanotubes which can absorb photons. These two tubular nanomotors could efficiently propel at very low concentration of H_2_O_2_ and without the addition of any surfactant. Under the irradiation of UV light, organic pollutants (e.g., Rhodamine B) can be decomposed by •OH generated during the photocatalytic reaction according to Equation (8). Escarpa and co-workers [80] reported that CdS/PANI/Pt micromotors can also be employed for the photodecomposition of organic pollutants (e.g., bisphenol A). Bisphenol A can be decomposed by •OH generated during the photocatalytic reaction described in Equation (9). The enhanced fluid mixing and transport caused by the fast locomotion of such micromotors, led to a greatly improved degradation yield (~100%) compared to that of their static counterpart structures (~20%).

Besides the degradation of the pollutants, the tubular Micro/Nanomotors were also widely used to remove the pollutants from water. Wang and co-workers [81] demonstrated that ZrO-graphene/Pt tubular micromotors could selectively capture and remove nerve agents from the environmental matrices. In this work, the graphene layer acted as nucleation sites for the growth of nano-sized ZrO and Pt particles. The nano-sized ZrO could selectively interact with the phosphate groups via acid-base Lewis interaction and thus effectively capture nerve agents and enrich phosphopeptides. Compared to their static counterparts, the greatly increased fluid transport caused by the motile micromotors led to a 15-fold faster removal remediation. The same group [34] demonstrated that the tubular micromotors can be utilized for the removal of oil from the polluted water (Figure 4C). For this application, the outer surface of tubular Au/Ni/PEDOT/Pt micromotors was functionalized with a long chain of alkanethiols via covalent conjugation to form a hydrophobic monolayer. Efficient motion of the tubular micromotor obviously facilitated the interaction between oil droplets and the alkanethiol chains. As-prepared tubular micromotors were able to load and transport multiple small olive oil droplets from the solution, while their corresponding static counterparts could not pick up such droplets. Apart from organic pollutants, heavy metals originated from various human industrial activities also do great harm to living systems. Hence, it is essential to develop efficient and inexpensive methods to capture and remove them from waste water [80,82,83,84]. For this reason, Sánchez and co-workers [82] reported the tubular GO/Ni/Pt micromotors for the efficient removal and recovery of Pb^2+^ ions from waste water (Figure 4D). Owing to the abundant oxygen moieties on GO nanosheets, the Pb^2+^ ions could spontaneously be absorbed into the outer layer of such tubular micromotors. In this case, mobile GO-micromotors could remove Pb^2+^ ten times more efficiently than non-motile GO-micromotors, cleaning water from 1000 ppb down to below 50 ppb in 60 min. After accomplishing decontamination of Pb^2+^ ions, these tubular micromotors could be easily removed from the aqueous solutions by a magnet. Subsequently, by adjusting the pH of solutions to acidity, the adsorbed Pb^2+^ ions would be released from the tubular micromotors, allowing them to be recycled and reused for further decontamination processes. Escarpa and co-workers reported [80] that tubular ZnS/PANI/Pt micromotors could remove Hg^2+^ ions from contaminated solutions. By immersing the tubular ZnS/PANI/Pt micromotors into contaminated water, Hg^2+^ ions were able to replace the Zn^2+^ ions in ZnS nanoparticles through cation exchange (Equation (10)). When ZnS was successfully converted into HgS, the solution color became bright yellow, which can be used to monitor the pollution of Hg^2+^ ions. Similarly, the enhanced fluid mixing and transport caused by the fast locomotion of these micromotors, led to a greatly improved removal yield (~100%) compared to that of their static counterpart structures (~20%).

Fe^2+^ + H_2_O_2_ → Fe^3+^ + OH^−^ + •OH (6)

C_6_H_5_NO_3_ + NaBH_4_ + H_2_O → C_6_H_7_NO + NaBO_2_ + H_2_O + H_2_ (Pd as a catalyst) (7)

organic pollutant + •OH → CO_2_ + H_2_O (8)

BPA + •OH → organic acids → CO_2_ + H_2_O (9)

ZnS + Hg^2+^ → HgS + Zn^2+^ (10)

Water remediation conducted by tubular Micro/Nanomotors has been proved to be efficient and environmentally friendly in test tubes. However, there still exist many challenges for future practical applications. For example, various chemical pollutants in the waste water will poison the Pt layer, which greatly affects the motion behaviors of these tubular Micro/Nanomotors. In addition, how to collect these tubular Micro/Nanomotors from the solutions after finishing their missions is still a tricky question.

### 4.2. Sensing

Owing to the unique propulsion mechanisms, the bubble-propelled tubular Micro/Nanomotors can locomote effectively in high ionic-strength media and relevant biological fluids. In addition, various functional units can be modified on the inner or outer surface of tubular Micro/Nanomotors. Therefore, the tubular Micro/Nanomotors hold considerable promise for various sensing applications in both environmental and biomedical fields.

#### 4.2.1. Environmental Sensing

**Figure 5:**
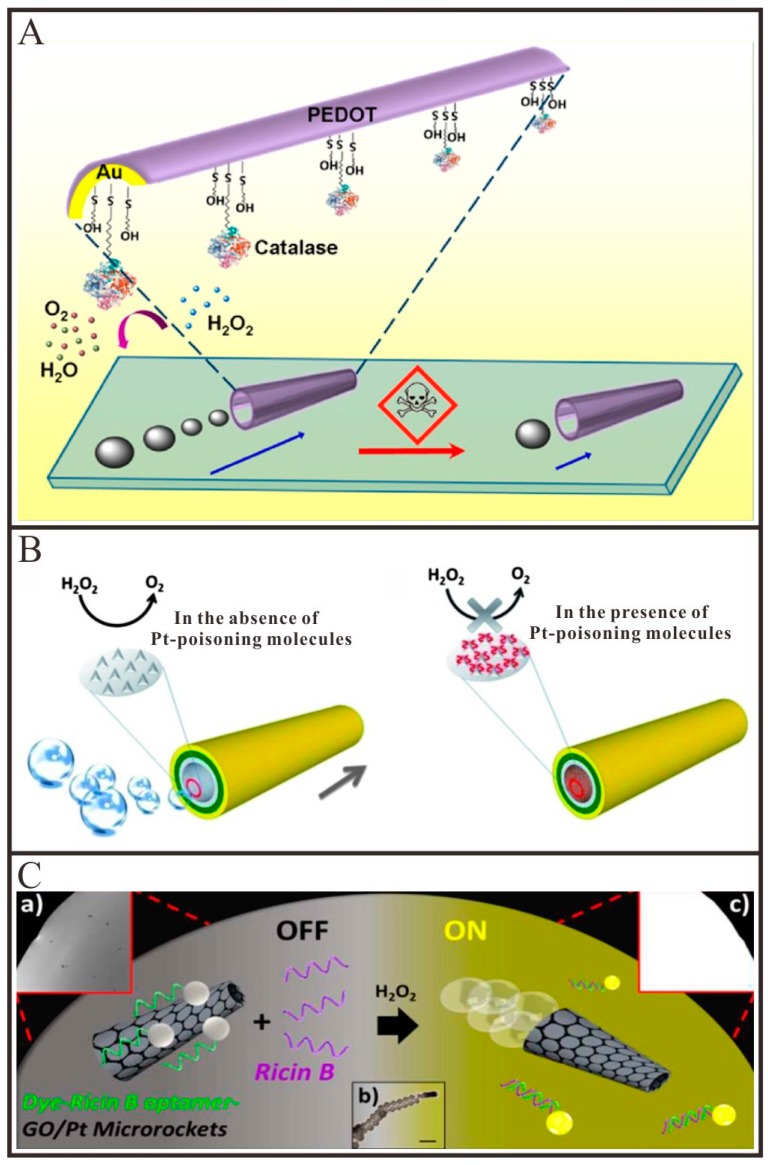
Representative examples of tubular Micro/Nanomotors for environmental sensing. (**A**) Schematic illustration of the pollutant effect on the micromotor locomotion speed through inhibition of the catalase biocatalytic layer (adapted from [39]); (**B**) Poisoning of the Pt-based micromotors with small molecules containing sulphur (adapted from [85]); (**C**) In vitro “off-on” fluorescent detection of ricin-B toxin by FAM-Ricin B aptamer-modified rGO/Pt micromotors (adapted from [91]).

In recent years, tubular Micro/Nanomotors have been demonstrated to have the capability to sense or monitor the water quality. The speed of the tubular Micro/Nanomotors is commonly used as a signal output for these sensors. Wang and co-workers [39] reported that the motion behavior of tubular PEDOT/Au-catalase micromotor can be used to test the water quality in the presence of aquatic pollutants (Figure 5A). A broad range of contaminants would affect the activity of catalase and thus impair the locomotion and survival time of these tubular micromotors. By monitoring the movement speed of the tubular micromotors, the water quality can be directly assessed. Pumera and co-workers [85] demonstrated that important extracellular thiols as well as basic organic molecules could significantly hamper the motion of Pt catalyzed tubular micromotors, prepared by both rolled-up and electrodeposition approaches, due to the poisoning of the catalytic Pt surface used to decompose H_2_O_2_ or the quenching of the hydroxyl radicals used to generate O_2_ (Figure 5B). The same team [86,87,88] also demonstrated that electrolytes such as Na^+^, K^+^, Ca^2+^, Cl^−^, SO_4_^2−^ and phosphates, uric acid and blood proteins such as bovine serum albumin, beta-globulin and glucose oxidase enzymes could hamper the mobility of Pt catalyzed tubular micromotors and be detected at small concentrations in the solution by monitoring the speed changes of corresponding micromotors. In order to clarify the influence of real-world environments on the motion of catalytic bubble-propelled micromotors, Pumera et al. [89] exposed the Cu/Pt bimetallic tubular micromotors to various types of water, including tap water, rain water, lake water and sea water, investigating their behaviors under real world conditions. They observed that the viability and mobility of such micromotors were strongly influenced by different water samples. The results were shown that there was a distinct negative correlation between the mobility of the micromotors and the ion content of the water found in real environments. Gao et al. [90] reported that the electrodeposited PEDOT/Pt tubular micromotors displayed remarkably high speeds in fuel-enhanced raw serum, apple juice, sea water, lake water and river water samples. The different results presented by the two research groups have not been found out and may arise from the difference in the material composition of the two micromotors.

Besides the speed of tubular micromotors, the fluorescence also can be utilized as the signal output [83,91,92]. Wang and co-workers [91] described a tubular micromotor-based sensor for the real-time detection of ricin B toxin in the surrounding environment. As depicted in Figure 5C, when the dye-aptamer adsorbed on the surface of graphene oxide via the π-π interaction, the fluorescence of the dye can be quenched by graphene oxide. While in the presence of ricin B, the dye-aptamer will release from the surface of graphene oxide owing to the specific binding between the ricin B and its aptamer, which resulted in the recovery of the fluorescence of the dye. By monitoring the fluorescence intensities of the solution, the concentration of ricin B can be real-time detected.

Tubular catalytic Micro/Nanomotors-based sensors have shown great potential for environmental monitoring. However, since a variety of species in waste water will affect the motion behaviors of tubular Micro/Nanomotors, the selectivity of those sensors which utilized the speed as the signal output will be not good.

#### 4.2.2. Biosensing

**Figure 6:**
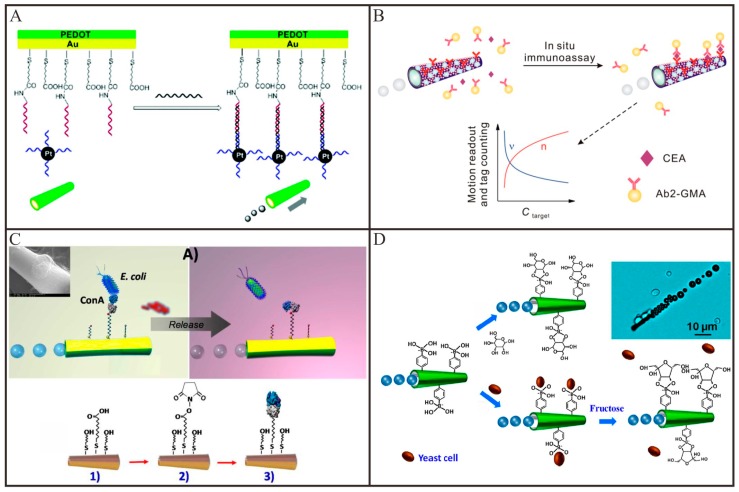
Representative examples of tubular Micro/Nanomotors for biosensing. (**A**) The principle for DNA detection by introducing Pt nanoparticle–DNA conjugate to the microtube via specific DNA hybridization (adapted from [94]); (**B**) Use for in situ immunoassay of protein biomarker via motion readout and tag counting (adapted from [98]); (**C**) A ConA-modified micromotor for selective isolation, transport and release of the target bacteria (adapted from [101]); (**D**) Schematic illustration of the PAPBA/Ni/Pt micromotor and its “on-the-fly” interaction with glucose (top) and yeast cell (bottom), along with triggered (fructose-induced) release of the cell (adapted from [33]).

Different from other micro/nanomaterials, the bubble-propelled tubular Micro/Nanomotors are able to autonomously locomote in biological liquids and thus provide an alternative method to isolate and detect biological analytes, such as nucleic acids, proteins, bacteria and living cells in unprocessed samples [40]. For example, Wang and co-workers [93] demonstrated that the tubular micromotor, which was modified with single-strand DNA on its outer surface via Au-S interaction, can selectively capture the target DNA from the raw biological samples and transport them to a clean location for subsequent analysis. Nguyen and Minteer [94] presented a novel DNA biosensor based on a tubular PEDOT/Au micromotor by using the speed as the signal output. As shown in Figure 6A, in the presence of the target DNA, the report DNA-modified Pt nanoparticle was captured to the inner surface of the tubular micromotors through the sandwich DNA hybridization. With the increased concentration of target DNA, the Pt catalyst nanoparticles attached to the inner wall will gradually increase, so the speed of the tubular micromotor will spontaneously increase. Recently, Wu and co-workers [95] demonstrated an efficient catalase-powered micromotor for the detection of target DNA. A multi-layer DNA and catalase were successively assembled on the inner surface of the tubular micromotor with the help of hybridization chain reaction. In the presence of target DNA, the sensing unit would hybrid with target DNA and release the multi-layer DNA as well as the multi-catalase, resulting in a decrease of the motion speed. Therefore, motion speed of such tubular micromotor-based biosensor could be used to roughly show the concentration of target DNA.

Motion-based design is not limited to sense DNA in biomedical field and it can also be extended to isolate and detect other biomacromolecules such as proteins [96,97,98,99,100]. For this purpose, Wang and co-workers [96] demonstrated a molecularly imprinted polymer-based tubular micromotor for the selective capture and transport of avidin. The recognition sites contained in the molecularly imprinted polymers were able to selectively capture the avidin from raw serum and saliva samples and transport them to predetermined destination. The same group [97] also demonstrated an aptamer-modified tubular micromotor for the isolation of thrombin from complex biological samples. This tubular micromotor can selectively capture the thrombin via the specific interaction between thrombin and its aptamer modified on the outer surface of tubular micromotors. The captured thrombin can be released by adding ATP which can bind and displace the immobilized mixed thrombin-ATP aptamer in 20 min. From isolation to detection, Wu and co-workers [98] fabricated a tubular micromotor-based biosensor for the detection of cancer biomarker, such as carcinoembryonic antigen (CEA). As depicted in Figure 6B, in the presence of CEA, the antibody-modified microspheres can be captured to the surface of tubular micromotors, leading to the decrease of the speed of tubular micromotors. The speed of the micromotors or the number of the microspheres conjugated on the micromotor can be used to monitor the concentration of the target proteins. Such microsensors can conveniently distinguish the concentration of CEA in a range of 1–1000 ng/mL and the whole detection procedure for protein target can be completed in 5 min without any washing and separation step. However, the speed of the tubular micromotor is influenced by various factors, thus those biosensors based on the speed as the signal output will be instable. Wang and co-workers [100] demonstrated a molybdenum disulfide (MoS_2_)-based tubular micromotor for thrombin detection by using the fluorescence as the signal output. The fluorescent dye-modified thrombin aptamer was first immobilized on the surface of MoS_2_ via π-π interactions. The fluorescent signal of the dye was rapidly quenched through the Förster resonance energy transfer (FRET) between the MoS_2_ and the fluorescent dye. The continuous movement of the receptor-functionalized tubular micromotors resulted in the self-mixing and enhanced contact with the thrombin. Upon the interaction, the fluorescent dye modified aptamer can specifically recognize the thrombin and lead to the release of the fluorescent dye modified aptamer from the surface of tubular micromotor. Thus, the fluorescent signal will be recovered.

Apart from biomacromolecules, self-propelled tubular micromotors can also be used to capture and release bacteria and living cells. Wang and co-workers [101] reported a lectin-modified tubular micromotor for the isolation of bacteria (*Escherichia coli*). As shown in Figure 6C, the concanavalin A (ConA) lectin modified on the outer surface of tubular micromotors can be utilized to recognize *Escherichia coli* and thus capture them from the clinical and environmental samples. By moving the tubular micromotors to a low pH-solution, the captured bacteria were released from the tubular micromotors owing to the dissociation of the sugar-lectin complex. An immuno-micromotor-based approach for in vitro isolation of circulating tumor cells was also explored by the same group [102]. By recognizing certain antigenic surface proteins such as CEA expressed on circulating tumor cells, these monoclonal-antibody-functionalized micromotors could selectively bind to target circulating tumor cells and then effectively transport them in phosphate-buffered saline and serum. Afterwards, the same team [33] presented a carbohydrate-sensitive tubular micromotor for the isolation monosaccharides and yeast cells. As imaged in Figure 6D, the outer surface of tubular micromotors was functionalized with boronic acid which can be used to selectively recognize monosaccharide. Thus, these boronic acid-modified tubular micromotors can be employed to bind and transport yeast cells which contain sugar residues on their wall. By the addition of fructose, the captured yeast cells can be release via a competitive sugar binding.

By integrating the sample pretreatment and subsequent detection in the same solution, tubular micro/nanomotor-based biosensors are simple and fast compared to conventional analytical methods [103,104,105]. The speed or the fluorescence signal of single tubular micro/nanomotor are generally used as the signal output, thus such biosensors can be utilized to the in-situ detection of the target analytes. However, there are still some issues for these biosensors. For example, the speed of Pt-based tubular Micro/Nanomotors is hampered in some biological media (e.g., proteins-rich media) [85]. Thus, the biosensors based on the speed as the signal output are instable. This can be alleviated by covering the Pt layer with a protective layer, such as MnO_2_ layer [106] or a polymeric layer [107]. Besides, the sensitivity of these tubular Micro/Nanomotors-based biosensors is very low. Thus, apart from the speed and fluorescence, other sensitive signal output should be explored to improve their sensitivity.

### 4.3. Active Drug Delivery

Over the past several decades, a large number of nanomaterial-based carriers have been employed for drug delivery. However, because of the lack of the capabilities of self-propulsion and controllable navigation, the drug delivery efficiency of these nanocarriers is still very low (<5%) [108]. Compared to the above-mentioned nanocarriers, Micro/Nanomotors are able to perform controlled navigation to targeted locations under physiological conditions and environments, as well as have the potential to rapidly transport and deliver therapeutic payloads directly to disease sites, thereby improving the therapeutic efficacy and reducing systemic side effects of highly toxic drugs. Thus, they represent a new and attractive class of delivery carriers that could potentially revolutionize drug delivery systems [109,110]. Among them, tubular Micro/Nanomotors have received great attentions owing to their strong thrust in biofluids. In the following subsection, we summarize the recent developments of in vitro and in vivo active drug delivery by tubular Micro/Nanomotors in detail.

**Figure 7:**
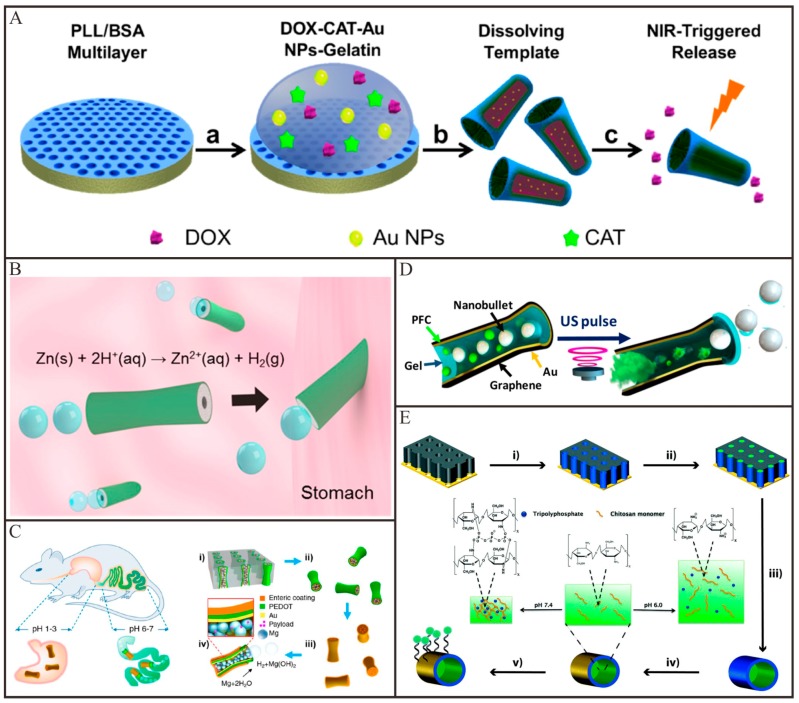
Representative examples of tubular Micro/Nanomotors for active drug delivery. (**A**) Fabrication and light-triggered drug release process of (PLL/BSA)_10_-DOX-CAT-AuNPs-Gelatin micromotors (adapted from [41]); (**B**) Schematic representation of the in vivo propulsion and tissue penetration of zinc-based micromotors (adapted from [112]); (**C**) Schematic illustration of in vivo operation of the enteric Mg micromotors for propulsion and fluorescent images of localized delivery to the gastrointestinal tract (adapted from [113]); (**D**) Schematic illustration of the firing of nanobullets from the microcannon-structured motor by the spontaneous PFC vaporization upon application of US pulse (adapted from [114]); (**E**) modified Ni nanotube motors for active drug delivery (adapted from [60]).

Chemically/biochemically propelled tubular Micro/Nanomotors have been widely explored for active drug delivery and tremendous progresses have been made in the past few years. The first example by using a tubular nanomotor for drug delivery was demonstrated by He and co-workers [75]. In this work, the tubular nanomotors were fabricated by a nanoporous template-assisted LbL assembly. Fluorescent anticancer drug doxorubicin (DOX), magnetic Fe_3_O_4_ nanoparticles and catalytic Pt nanoparticles were encapsulated into the multilayer wall of this tubular polymeric nanomotor, respectively. Under the guidance of magnetic fields, the nanomotors can autonomously transport and steer to the target Hela cells. After the nanomotors were attached to the outer surface of the target HeLa cell, the DOX molecules encapsulated in the nanomotors can be released through the ultrasound irradiation. Wang and co-workers [100] fabricated molybdenum disulfide (MoS_2_)/Pt tubular micromotors for active drug delivery. DOX was loaded onto the MoS_2_ layer of tubular micromotors via π-π stacking and hydrophobic interactions. At lower pH, DOX will release from MoS_2_ surface owing to the protonation of the amino group on DOX. A high concentration of H_2_O_2_ was considered to be incompatible with living organisms so that scientists have paid much attention to seek more active catalysts than Pt to replace Pt. For example, He and co-workers [41] demonstrated a catalase-based tubular micromotor for drug delivery. As depicted in Figure 7A, these tubular micromotors were fabricated by a template-assisted LbL technique, following the integration of a thermal-sensitive gelatin hydrogel in which catalase, Au nanoparticles, magnetic nanoparticles and DOX were encapsulated. The experimental results demonstrated that this catalase-based tubular micromotor can drive at a very low concentration of H_2_O_2_. With the magnetic guidance, the DOX-loaded tubular micromotors can transport to the targeted cancer cell. Upon the irradiation of NIR light, Au nanoparticles will absorb energy and thus heat the gelatin hydrogel. After the gelatin hydrogel melted, the DOX will be released from the microtubes and subsequently kill the surrounding cancer cells. In order to totally eliminate the effect of H_2_O_2_, alternative fuels should be used to drive tubular motors. Wang and co-workers reported [111] an acidic fuel-driven tubular micromotor for combinatorial delivery and release of multiple cargos. The double-conical tubular Zn micromotors were prepared by template-electrodeposition and fully loaded silica and Au nanoparticles through particle-infiltration techniques. When introduced in acidic fuel media, these tubular Zn micromotors were propelled by ejecting H_2_ bubbles. When the Zn body was dissolved, the encapsulated cargos were released autonomously from the micromotors. More recently, chemically propelled tubular micromotors have been moved to the living animal models for active drug delivery. For example, Wang and co-workers [112] firstly applied acidic fuel-driven tubular micromotors for active drug delivery in the stomach of living mice. Owing to the harsh acidic environment in stomach, the Zn-based tubular micromotors fully loaded with cargos can display efficient propulsion. As shown in Figure 7B, these Zn-based tubular micromotors led to a dramatically increased retention of cargos in the stomach of mice. Afterwards, the same group [113] demonstrated that the Mg-based tubular micromotors with enteric coating can be utilized for drug delivery in the gastrointestinal tract (Figure 7C). The enteric coating was stable in acidic conditions but soluble in neutral or alkaline intestinal fluid. The enteric coating on the outer surface of tubular micromotors was used to position in the gastrointestinal tract, while the Mg particles loaded in cavity of tubular micromotors were used to propel in intestinal fluid. By tailoring the thickness of the enteric coating, they can tune the time required to dissolve the polymer layer, thereby controlling the distance that the tubular micromotors can travel in the gastrointestinal tract before their propulsion was activated. Upon activation, the tubular micromotors will propel and penetrate into the local tissue and remain there to release payloads.

Compared with the so far chemically/biochemically propelled Micro/Nanomotors, external field-propelled Micro/Nanomotors are more suitable for active drug delivery. This is because of the good biocompatibility, long sustainability and robust motion control. Recently, tubular Micro/Nanomotors propelled by ultrasound and magnetic fields have also been explored for active drug delivery. Wang and co-workers [114] demonstrated that the ultrasonically triggered tubular micromotors are able to loading and firing multiple cargos. As imaged in Figure 7D, silica/fluorescent microspheres and PFC emulsions were fully loaded in hollow conically shaped microtubes. Hoop and co-workers [60] fabricated a multifunctional magnetically propelled micromotor for active drug delivery. As depicted in Figure 7E, this micromotor consisted of a magnetic Ni microtube, which can be propelled by means of external magnetic fields. A pH-responsive chitosan hydrogel was functionalized in the inner cavity of Ni microtube, which served as a matrix to load drugs. By altering the pH values to acidity, the loaded drug will be release from micromotors owing to the swelling or dissolution of the hydrogel. A fluorescence tag (FITC labeled thiol-ssDNA) was conjugated on the outer surface of Ni microtube, which was used to trace the tubular micromotors. These magnetically propelled tubular micromotors can precisely navigate to targeted cells and release the encapsulated drugs. More recently, a sperm-biohybrid micromotor has been developed for active drug delivery [115]. This micromotor comprised a motile sperm that served as the propulsion source and drug (DOX) carrier and a 3D printed four-armed microtube, used for the magnetic guidance and mechanical release of the drug-loaded sperm cell in the desired area. The drug delivery occurred when the tubular microstructures bend upon pushing against a tumor spheroid and the sperm squeezed through the cancer cells and fused with cell membrane, thus minimizing toxic effects and unwanted drug accumulation in healthy tissues. These sperm-hybrid micromotors might have the potential application for gynecologic cancer and other diseases in the female reproductive tract treatment.

The proof-of-concept studies of tubular Micro/Nanomotors for active drug delivery have received good performance in vitro and in vivo. To extend these tubular Micro/Nanomotors to practical applications, several key issues, such as the size of tubular motors, the biocompatibility of the energy sources and the controllability of the motion behaviors etc., should be addressed by future research.

### 4.4. Precise Surgery

Unlike their macroscopical counterparts, Micro/Nanomotors with a strong driving force and deep penetration ability can potentially navigate throughout human body and operate in many hard-to-reach tissue locations [116,117]. Thus, they would have great promise to assist doctors to operate various minimally invasive surgeries in living body with high precision and flexibility. In this subsection, we summarize the recent advances of precise surgery based on tubular Micro/Nanomotors.

**Figure 8:**
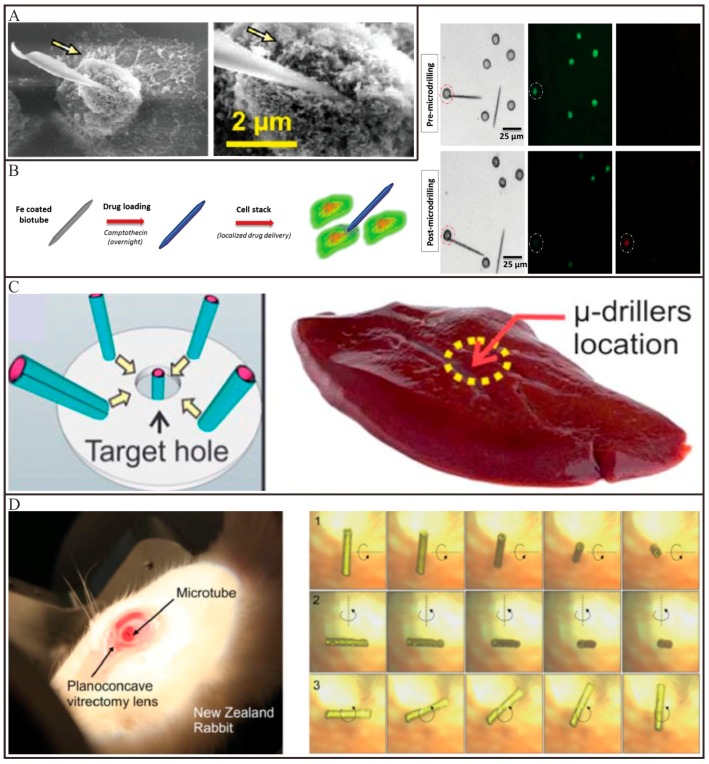
Representative examples of tubular Micro/Nanomotors for precise surgery. (**A**) SEM images of the guided catalytic InGaAs/GaAs/(Cr)Pt micromotors before and after drilling a single cell (adapted from [118]); (**B**) Schematic representation of imparting magnetic and drug delivery properties to the biotube, as well as fluorescent images of live cells (green) and dead cells (red) before and after microdrilling, respectively (adapted from [43]); (**C**) Schematic image showing the fuel-free motion of the micromotors towards the center of magnetic field and the drilling operation on pig liver tissue (adapted from [44]); (**D**) A living New Zealand rabbit eye with a micromotor and rotation of the micromotor around three axes at a rotating magnetic field in the vitreous humor (adapted from [119]).

Recent advances in tubular Micro/Nanomotors have shown considerable feasibility for applying these tiny devices to precise surgery. Solovev and co-workers [118] utilized a catalytically propelled InGaAs/GaAs/(Cr)Pt tubular micromotor to drill into biomaterials such as those constituting HeLa cells. By tuning the tips of microtubes, the tubular micromotors can perform different movement. An asymmetrically tubular micromotor with a sharp tip could move in a corkscrew-like trajectory in the H_2_O_2_ solutions, which can drill deeply into the fixed HeLa cells, as imaged in Figure 8A. Owing to the high concentration of H_2_O_2_ involved, these tubular micromotors are unsuitable for practical applications. Cai and co-workers [59] demonstrated that a magnetically propelled Ni-embedded carbon nanotube can be utilized to penetrate into cell membranes. With the combination of rotating and static gradient magnetic fields, the carbon nanotube can spear and penetrate deeply into cells on a substrate. DNA plasmids loaded on the surface of the nanotubes subsequently released into targeted cells. Srivastava and co-workers [43] reported a magnetically propelled biotube for single-cell surgery and drug release. The biotubes were extracted from the *Dracaena* sp. Plant and coated with a Fe layer via an e-beam deposition process to incorporate magnetic control (Figure 8B). These biotubes possessed two functions that are the creation of cellular incision together with site-directed drug delivery. Apart from operating on single cell levels, the magnetically propelled tubular micromotors can also be operated in tissues or even a living animal. For example, a tubular Ti/Cr/Fe microdriller with a sharp tip was used to operate surgery in porcine liver tissue ex vivo by Sánchez and co-workers [44]. With the propulsion of an external rotational magnetic field, this tubular microdriller can embed inside the porcine liver tissue (Figure 8C). Pané and co-workers prepared a tubular CoNi micromotor for performing surgery in the eye of a living rabbit. As illustrated in Figure 8D, the tubular micromotor can be controlled wirelessly in the central vitreous of the rabbit eye with a rotating magnetic field [119]. These implantable tubular micromotors have the potential for targeting diseases in confined spaces of the human body.

The above-mentioned studies have demonstrated that tubular Micro/Nanomotors have great potential to operate precision surgery in vitro or even in living animal models. However, this is still an alluring but unmet goal remaining for biomedical researchers. More efforts should be done to extent these surgical tubular Micro/Nanomotors to practical applications.

## 5. Conclusions and Outlooks

Over the past decades, remarkable progress has been made in both understanding and applying tubular Micro/Nanomotors. Up to date, various energy sources, such as chemical/biochemical reactions, external fields and motile microorganisms, have been used to drive tubular Micro/Nanomotors. With the development of nanotechnology, different fabrication techniques, which mainly include rolled-up and template-assisted methods, have been developed to design and fabricate tubular Micro/Nanomotors with different sizes (the length in tens of nanometers to hundreds of micrometers) and special functionalities. Owing to their unique properties, such as the strong driving force and easy surface functionalization, tubular Micro/Nanomotors have been widely utilized for environmental and biomedical applications in terms of water remediation, sensing, active drug delivery and precise surgery. More recently, one important application of tubular Micro/Nanomotors in food-related fields is also emerging [120,121]. Although great advances have been made for tubular Micro/Nanomotors, several issues are still yet to be prior addressed to realize future practical applications. For example, Schmidt and co-worker [122] pointed out that medical micromotors need better imaging and control. Here, we generally put forward three challenges combining reported literatures and our own thoughts. First, the motion behaviors of tubular Micro/Nanomotors should be further improved. To date, most tubular Micro/Nanomotors are propelled by H_2_O_2_, which, however, is considered to be incompatible with living organisms at a high concentration. Although bio-friendly fuels, such as acids or urea, can be used as alternative fuels to power tubular Micro/Nanomotors, these tubular Micro/Nanomotors suffer from a weak driving force or limited lifetime. Second, facile and cost-effective fabrication techniques should be developed toward parallel mass production of tubular Micro/Nanomotors. Recently, the rolled-up methods are always dependent on complicated fabrication process and expensive facilities, while the template-assisted methods are difficult to tune the shape or size of the products. The geometry and morphology of the tubular Micro/Nanomotors play a vital role for their motion behaviors. The uniformity of the structures and reproducibility of the methods are of great importance for the practical applications. Thus, quality control cannot be ignored when simplifying the fabrication procedures. The fabrication methods of microtubes developed by our group [50,123] provide a good choice. Third, more efforts should be done to improve the performance of the tubular micro/nanomotor-based tools for practical applications in the future. For environmental applications, tubular Micro/Nanomotors have been demonstrated to be powerful in test tubes. However, how to move these tiny devices from test tubes to real environments is still a big challenge. For tubular Micro/Nanomotors-based sensors, other forms of signal output should be explored to improve their analytical performances (e.g., sensitivity or selectivity) which are inferior to the conventional methods. For active drug delivery and precise surgery, a large number of motors should cooperate and communicate with each other to accomplish tasks. We envision that above challenges can be gradually addressed, eventually expanding the horizon of tubular Micro/Nanomotors in various fields.
